# Darbepoetin and Infantile Hemangioma in Premature Infants

**DOI:** 10.3390/jcm15166445

**Published:** 2026-08-20

**Authors:** Irit Shoris, Arieh Riskin, Livnat Sharkansky, Rasha Zoabi-Safadi, Adir Iofe, Arina Toropine, David Bader, Ayala Gover

**Affiliations:** 1 Neonatal Intensive Care Unit, Bnai Zion Medical Center, Haifa 31048, Israel; shirit94@gmail.com (I.S.); arik.riskin@gmail.com (A.R.); livish@windowslive.com (L.S.); rashazoabi@gmail.com (R.Z.-S.); adir.iofe@gmail.com (A.I.); arina790@gmail.com (A.T.); davidbade@gmail.com (D.B.); 2 Ruth and Bruce Rappaport Faculty of Medicine, Technion Israel Institute of Technology, Haifa 32000, Israel

**Keywords:** preterm, erythropoiesis-stimulating drugs, darbepoetin

## Abstract

**Objectives**: An association between erythropoietin (Epo) administration and infantile hemangiomas (IH) in preterm infants was previously described; however, scarce data exists on a newer drug, darbepoetin (Darbe) and IH. We aimed to determine whether Darbe treatment was associated with IH in our preterm population. **Methods**: Retrospective observational cohort study including a consecutive sample of premature babies born < 32 weeks at a single tertiary center between the years 2012–2023. We compared the rate of IH with Epo exposure, Darbe exposure and no exposure. **Results**: The cohort comprised 390 infants: 262 infants with no exposure, 36 infants with Epo exposure, and 92 infants with Darbe exposure. Exposed infants had significantly lower birth weight and gestational age compared to non-exposed infants. No differences were found in gestational age or birth weight between the two drug exposure groups. The rate of IH was 6.8%, 8.3% and 18.4% in the no exposure, Epo exposure and Darbe exposure groups, respectively. The rate of IH in the Darbe group was significantly higher compared to the non-exposed group (*p* = 0.003), and Darbe exposure was found to be significantly correlated with IH in a multiple logistic regression model (OR 3.07, 95% CI 1.5–6.2; *p* = 0.002). However, after adjusting for gestational age or birth weight, this correlation was no longer significant. **Conclusions**: Darbe exposure was associated with a higher rate of IH than no exposure in this cohort, but the association did not persist after adjustment for maturity, and the design does not allow us to distinguish the effect of Darbe from the effect of prematurity itself. Further prospective studies are needed to ascertain these findings.

## 1. Introduction

Anemia of prematurity is a common complication in preterm infants, related to impaired erythropoiesis due to immaturity, along with frequent blood sampling [1]. Blood transfusions have been the primary means of treatment; however, they cause suppression of erythropoiesis and may increase the risk for complications. Previous data suggests an association between blood transfusions and necrotizing enterocolitis (NEC), sepsis, bronchopulmonary dysplasia (BPD), severe retinopathy of prematurity (ROP), and periventricular leukomalacia (PVL) [2,3].

Since the late 1980s, treatment with recombinant human erythropoietin (Epo) has been extensively studied as a strategy to minimize red blood cell transfusions in preterm infants [4,5,6,7,8,9]. In the last decade, a newer genetically engineered form of erythropoietin containing 5 amino acid changes called darbepoetin alfa (Darbe) has been in growing use in preterm infants [10]. It effectively stimulates erythropoiesis and has a half-life 3 times longer than Epo.

One concern regarding erythropoiesis-stimulating drugs in preterm infants has been a relationship between the administration of Epo and the appearance of infantile hemangiomas [11,12]. Infantile hemangiomas (IH) are benign vascular tumors that develop after birth until early childhood [13]. The growth of small blood vessels may be related to rapid angiogenesis through the proliferation of the vascular endothelium [14]. In about 10–20% of cases, excessive vascular growth in problematic regions can block the field of vision, airways or interfere with feeding [13].

While some studies examined the association between Epo and IH, to our knowledge, there are no data on the relationship between Darbe treatment and IH in premature infants. In 2015, our department moved from routine Epo treatment, which required 3 weekly injections, to Darbe, which required a single weekly injection.

Our aim was to examine whether Darbe treatment was associated with IH in our preterm population.

## 2. Methods

This was a retrospective cohort study including premature babies born < 32 weeks of pregnancy at Bnai Zion Medical Center, Haifa, between the years 2012–2023. The study was approved by the Bnai-Zion Research Ethics Board (approval no. 0108-23BNZ). Due to the retrospective nature of the study, informed consent was waived.

Data were collected from electronic medical records based on a query in the computerized registries “Chameleon” medical records software (Chameleon Medical Innovation Ltd., version 8.5.15.19514, Yakneam, Israel) and “Max 2000” medical records’ manager system (Max Software Ltd., version 5.71.61, Haifa, Israel). All premature infants born before 32 weeks of pregnancy from 01 January 2012 to 30 September 2023 were identified and the following data were retrieved: (1) Demographic data (e.g., gestational age (GA), birth weight (BW)) (2) maternal morbidity (3) Complications of prematurity (BPD, ROP, NEC, intraventricular hemorrhage (IVH)) (4) exposure to erythropoiesis stimulating drugs, number of blood transfusions, lowest and highest hematocrit value during hospitalization, number of reticulocytes 2 weeks after starting erythropoiesis stimulating treatment (5) documented hemangiomas during hospitalization until discharge.

All data were anonymized, encoded and saved electronically in a dedicated protected file. The patients were divided into 3 groups: (1) No exposure to erythropoiesis-stimulating drugs (2) Exposure to Epo treatment (3) Exposure to Darbe treatment.

The decision to begin treatment with erythropoiesis-stimulating drugs during the study period was made by the attending physician, guided by the general departmental protocols: Epo was recommended in stable preterm infants born < 32 weeks of gestation with a hematocrit < 35% after the first week of life, and was discontinued close to discharge, usually at 35 weeks postmenstrual age (PMA). In sick infants, especially those who had received multiple blood transfusions in the first few days of life, the treatment was delayed or not administered at all. In practice, not all physicians prescribed Epo, and when used, it was primarily given to smaller and more immature preterm infants and rarely given beyond 30 weeks’ gestation. When the department transitioned from Epo to Darbe, the gestational age threshold was adjusted to <30 weeks to reflect the prevailing clinical approach already in use. Darbe was also typically given after the first week of life or to older premature babies who had a hematocrit < 35% during the first month of life until they reached 35 weeks postmenstrual age.

Epo (Erythropoietin alfa recombinant—Roche Diagnostics GmbH, Mannheim, Germany) was given subcutaneously at a dose of 400 units/kg/dose, 3 times a week. Darbepoetin alfa (Aranesp. Amgen Europe B.V., Breda, The Netherlands, importer—Medison Pharma Ltd., Petach Tikva, Israel) was given subcutaneously at a dose of 10 mcg/kg once weekly.

Our iron supplementation dosing for infants receiving erythropoietin-stimulating drugs was 5 mg/kg/day. Transfusion thresholds were guided by hematocrit, cardiorespiratory stability and the extent of respiratory support, following standard guidelines [15].

The diagnosis of IH was made and documented by any of the examining physicians during physical exam, and in cases of more than 3 lesions, liver ultrasound was performed.

Data were recorded in an Excel spreadsheet (Microsoft Office, Seattle, WA, USA) and statistically analyzed using SigmaPlot, version 11.0 (Systat Software Inc., San Jose, CA, USA) and Minitab^®^, version 16.2.2 (Minitab Inc., State College, PA, USA & Coventry, UK). Statistical analysis to determine whether there was a relationship between drug exposure and the appearance of IH as well as other associations included: descriptive statistics; Student’s *t*-test or Mann–Whitney (Rank Sum) U-Test for comparison of two continuous variables and one-way analysis of variance (ANOVA) or Kruskal–Wallis ANOVA on Ranks for more than two continuous variables, according to their distributions; and Chi-Square test or Fisher Exact test for comparisons of categorical variables. Multiple logistic regression analysis was used to examine together factors that could have affected the primary outcome. We incorporated into the model demographic and clinical parameters with *p*-value < 0.1 in the univariate analysis. Data for continuous and ordinal variables are presented as mean with standard deviation or median with interquartile range as appropriate. Categorical variables are presented as numbers and percentages. Statistical significance was defined as *p* < 0.05, and odds ratios with 95% confidence intervals are shown.

## 3. Results

**Figure 1:**
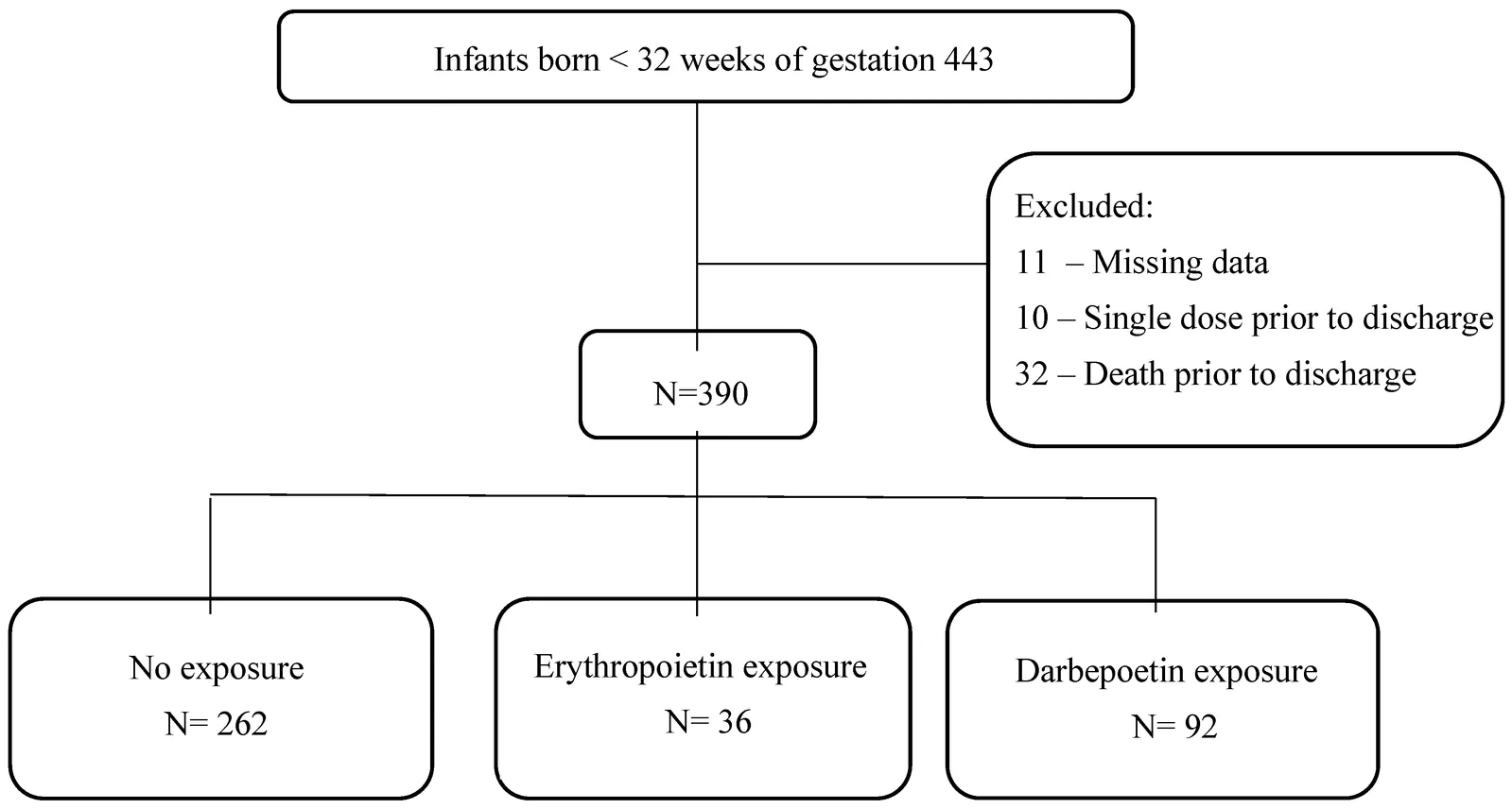
Cohort flow diagram.

A total of 443 patients born < 32 weeks of gestation during the study period were identified. After excluding infants who did not survive to discharge, cases with missing data, and cases with exposure to just one single dose of Epo/Darbe close to discharge, the final cohort comprised 390 infants (Figure 1).

There were 262 infants (67%) with no exposure to erythropoietin-stimulating drugs, 36 infants with Epo exposure, and 92 infants with Darbe exposure. Of all infants, there were 198 female (50.7%) and 192 male (49.2%), and there were no sex differences between the exposure groups. Drug exposure varied with gestational age. Almost all infants born between 23 and 26 weeks were exposed (34/36, 94.4%), more than half of the infants born at 27–30 weeks were exposed (80/139, 57.5%), and only a few of the infants born at 31–32 weeks were exposed (14/215, 6.5%). Therefore, exposed infants had significantly lower birth weight and gestational age compared to non-exposed infants (*p* < 0.001). No differences were found in gestational age or birth weight between the two drug exposure groups (Table 1). Due to the differences in gestational age and birth weight between non-exposed and exposed infants, all direct comparisons between these groups should be interpreted with caution.

Both exposure groups had significantly lower hematocrits and higher numbers of reticulocytes compared to non-exposed infants (*p* < 0.001), but no significant differences were found between the two exposure groups.

The rate of IH was significantly higher in the Darbe group compared to the non-exposed group (18.4% vs. 6.8%, respectively, *p* = 0.003), but did not reach significance compared to the Epo group (18.4% vs. 8.3%, respectively, *p* = 0.15). There was no significant difference between the Epo group and non-exposure group (8.3% vs. 6.8%, respectively, *p* = 0.98) (Table 2). When stratifying for gestational age, the rate of IH increased with decreasing gestational age (Table 3). The differences in rates of IH between the exposure groups for each stratified patient population were not statistically significant. In a sub-analysis of infants born <30 weeks of gestation, performed in order to confine the comparison to the gestational-age range in which these drugs were prescribed in our unit, the effect size was preserved but the association was no longer statistically significant (19.8% vs. 9.8%; OR 2.26, 95% CI 0.83–6.16; *p* = 0.16). In a multiple logistic regression model, any drug exposure was significantly correlated with the appearance of IH (OR 2.51, 95% CI 1.28–4.94; *p* = 0.008), but this correlation was no longer significant after adjusting for gestational age (OR 1.48, 95% CI 0.55–3.97; *p* = 0.442) or birth weight (OR 1.73, 95% CI 0.72–4.17; *p* = 0.222). To elucidate the specific effect of Darbe compared to Epo, a multinomial logistic regression model was used. Darbe exposure was significantly correlated with IH (OR 3.07, 95% CI 1.51–6.26; *p* = 0.002), and with having ≥ 3 IH (OR 6.02, 95% CI 1.48–24.60, *p* = 0.012). These associations were attenuated and no longer significant after adjustment for gestational age (OR 2.02, 95% CI 0.70–5.84; *p* = 0.195 and OR 2.60, 95% CI 0.35–19.32; *p* = 0.352, respectively) or birth weight (OR 2.20, 95% CI 0.88–5.52; *p* = 0.092 and OR 4.17, 95% CI 0.69–25.20; *p* = 0.120), and in a multivariable model including gestational age, birth weight, plurality and preeclampsia (OR 1.83, 95% CI 0.63–5.30; *p* = 0.263). Adjustment for duration of observation (length of stay) attenuated the association slightly (OR 2.67, 95% CI 1.14–6.26; *p* = 0.023), but it did not predict IH detection once gestational age was accounted for (OR 0.99 per 10 additional days, 95% CI 0.86–1.13, *p* = 0.85). Epo was not significantly correlated with IH (OR 1.2, 95% CI 0.3–4.4; *p* = 0.748).

Significantly more infants developed three or more lesions in the Darbe group compared to the no-exposure group (6.5% vs. 1.1%, *p* = 0.011), and in one case of multiple hemangiomas in the Darbe group, a large hepatic hemangioma was found. Among only those infants in whom a hemangioma had been detected, three or more lesions were present in 6/17 (35.3%) of darbepoetin-exposed infants versus 3/18 (16.7%) of unexposed infants (*p* = 0.26). Although this comparison did not reach statistical significance in the small number of affected infants, the direction of the difference was preserved.

As expected, both exposure groups had higher rates of complications of prematurity compared to the non-exposure group, due to gestational age and birth weight differences. Both exposure groups had comparable rates of NEC, days on high oxygen requirement, and number of RBC transfusions (Table 2). However, the Darbe group had a significantly higher rate of BPD compared to the Epo group (Table 2).

Within-group analyses in exposed infants with and without IH showed mostly similar characteristics and short-term outcomes of exposed infants (Table 4); however, in an exploratory analysis, ROP appeared more frequently in Darbe-exposed infants with IH than in those without IH (9/17, 52.9% vs. 18/75, 24.0%; *p* = 0.04). This comparison was one of multiple within-group comparisons performed in a small subgroup and was not corrected for multiplicity; it should be regarded as hypothesis-generating.

## 4. Discussion

In this retrospective observational study of preterm infants < 32 weeks of gestation, we found a higher-than-expected rate of hemangiomas in infants who were exposed to darbepoetin.

Infantile hemangioma is the most common benign tumor in infancy [16], characterized by proliferation of immature endothelial cells and occurring in 4–10% of infants by one year of age. In some cases, serious complications may occur, including bleeding, infections, ulcers, and vision or airway obstruction. Prematurity and low birth weight (LBW) are significant risk factors [16,17,18,19], along with female sex, multiple gestation and a family history of IH [16]. One study found that every 500 g decrease in birth weight increases the risk of IH by 40% [20]. Furthermore, the risk of hemangioma ulceration is elevated in preterm infants compared to infants born at term [21].

Erythropoiesis-stimulating drugs are commonly used in preterm infants to stimulate the production of red blood cells. Epo has been in use for over 20 years [5] and was previously shown to have a vasoproliferative effect on endothelial cells [22,23] and to induce angiogenesis in addition to erythropoiesis. This is thought to be mediated by vascular endothelial growth factor (VEGF), which is an essential regulator of fetal angiogenesis, and may trigger the development of hemangiomas in preterm infants [24,25]. An association between Epo exposure and the appearance of IH in preterm infants was previously described. Following a few case reports [26,27], a large retrospective cohort study of over 2500 preterm infants born between 23 and 36 weeks of gestation found a hemangioma rate of 16% in infants exposed to Epo [11] compared to an overall rate of 4% until discharge. While lower gestational age and birth weight increased the risk of developing hemangiomas, Epo was found to be an independent risk factor for the appearance of IH in a multivariate analysis after adjusting for these factors. Another case report described multiple cutaneous hemangiomas and diffuse hepatic hemangiomas developing in a preterm infant after just 3 weeks of Epo treatment for anemia of prematurity [12]. Conversely, a large randomized controlled trial of early high-dose erythropoietin given from 24 to 32 weeks for neuroprotection (PENUT trial) found a rate of 8% IHs in the Epo group and 6% in the placebo group (non-significant) [28], and in another randomized controlled trial of high-dose Epo, the rate of IH was 9.8% in the placebo group vs. 13.1% in the Epo group, and this difference was also non-significant [29]. Our rate of IH in the Epo group (8.3%) was comparable to the non-exposed group of older preterm infants, and is in agreement with the rate found in the PENUT trial.

Darbepoetin alpha is a newer erythropoiesis-stimulating agent with growing use in the preterm population due to its similar effectiveness in decreasing the number of blood transfusions and a longer half-life compared to erythropoietin, allowing for fewer doses [10]. To our knowledge, the association between Darbe and IH has not been studied.

We found a relatively high rate of IH (18.4%) in infants exposed to Darbe compared to the rates of IH reported in most other studies done in preterm infants until discharge, and exceeding the rate reported in Epo trials. One study of infants born ≤ 1250 g found a rate of 13.9% (n = 351) of infantile hemangiomas at the time of discharge [30]. A larger study of over 2500 preterm infants reported an overall rate of 4.3% of hemangiomas during NICU stay [11]. Interestingly, another study of over one million preterm infants < 32 weeks of gestation reported a strikingly lower rate of less than 0.5% IH at discharge [31]. Higher rates (up to 22.9%) were reported in studies following extremely preterm infants < 1000 g to 1 year of age [32,33].

Despite the significantly higher rate of IH with Darbe exposure compared to non-exposure, the statistical significance was lost in analyses that included stratifying and adjusting for gestational age or birth weight, probably because our group of non-exposed infants comprised older and larger preterms, in addition to insufficient sample size and lack of power. Within the Darbe-exposed group, gestational age and birth weight did not differ between infants who did and did not develop IH, although this cannot isolate an effect of Darbe, since exposure does not vary within this group. Lesion multiplicity was more frequent with Darbe exposure, and the direction of this difference was preserved, though without statistical significance, when the analysis was restricted to infants in whom a hemangioma had been detected. If a true association exists, we speculate that the longer half-life of Darbe may produce more sustained, rather than intermittent, erythropoietin-receptor stimulation, with correspondingly greater angiogenic potential.

Unlike previous studies demonstrating a higher risk of IH in females [11,16,20,34,35], we did not find sex differences between the groups with and without IH. There were also no statistically significant differences in birth weight, maternal age and multiple gestation between infants with and without hemangioma.

We found significant differences in several complications of prematurity between the non-exposed group and the two exposure groups, as expected due to the significant difference in their GA and BW. However, the rate of any BPD was higher in the Darbe group compared to the Epo group, which were of similar GA and BW. A recent meta-analysis of 14 studies assessing the effect of Epo on BPD reported no impact of Epo on BPD defined by oxygen requirements at 36 weeks post menstrual age [36]. However, unlike our finding, a randomized trial of Darbe vs. Epo and placebo in infants < 1250 g found no differences in BPD between the three groups, although sample size was relatively small [10]. Interestingly, a large randomized trial of Darbe vs. placebo in infants < 28 weeks of gestation [37] demonstrated a decrease in moderate-severe BPD (grade 2–3, but not grade 1—defined by newer criteria [38]) in infants exposed to Darbe compared to placebo, and this effect may have been mediated by fewer RBC transfusions in the Darbe group [39]. In our study, most infants had mild BPD (based on the previous definition of BPD [40]), and the rate of moderate/severe BPD, as well as the number of days on supplemental oxygen > 30% and the number of RBC transfusions, was comparable between the Epo and Darbe groups.

In an exploratory within-group analysis, ROP was more frequent among Darbe-exposed infants with IH than among those without IH. This observation is based on a small subgroup and requires confirmation, but it is consistent with the presumed common pathophysiology of these conditions [41]. Both IH and ROP are disorders of vascular proliferation. Retinal vascularization begins in the fetus at about 12 weeks of gestation and is completed around 36 to 40 weeks of gestation. In preterm infants, incomplete retinal vascularization at birth, along with relatively high oxygen exposure, may result in inhibition and later compensatory overgrowth of retinal vasculature, mediated by VEGF. IH progression is also characterized by a vasoproliferative phase speculated to follow local tissue hypoxia and interrupted vascularization, mediated by VEGF among other factors [41]. Both disorders respond to beta-2 blockade, which inhibits VEGF production. Some previous clinical data raised concern regarding an association between Epo administration and ROP [41,42,43], probably mediated by the angiogenic properties of Epo; however, other studies [10,28,44,45] did not show this association, and a study comparing Darbe to placebo showed comparable rates of ROP between the groups as well [10].

Previous studies demonstrated the co-occurrence of ROP and IH in preterm infants. One study found a significant relationship between the occurrence of ROP and IH in preterm infants < 32 weeks GA [41], and another large database study analyzing over 1 million newborn records found a significant association between ROP and IH after adjusting for GA and postnatal steroid use [31].

To our knowledge, this study is the first to specifically assess the association between IH and Darbe exposure in preterm infants. Our study has several limitations. Our exposed and unexposed groups did not overlap sufficiently in gestational age and observation periods to permit an unbiased comparison, and residual confounding cannot be excluded. Data on IH were available only to discharge, which probably limited the true rate since IHs frequently develop later during the first year of life. In addition, information regarding lesion location, size and need for treatment was unavailable, limiting insight into their clinical significance. Due to the retrospective and observational nature of this study, non-exposed infants were of higher gestational age at birth than both exposure groups, introducing selection bias regarding the rate of IH and complications of prematurity; since more immature infants remained hospitalized longer and were therefore examined more often, this could have introduced surveillance bias favoring detection of IH in the more premature, drug-exposed infants. IH was diagnosed by the treating physicians during routine examinations rather than through a standardized screening protocol, which introduces interobserver variability and detection bias due to examinations performed by different physicians.

In conclusion, we found a higher than expected rate of IH in preterm infants exposed to Darbe. Further prospective investigations are needed, with standardized dermatological assessment, prespecified documentation of lesion number, site, size and treatment, and sufficient sample size to distinguish the effect of Darbe from the effect of prematurity itself. In the meantime, these observations warrant increased clinical awareness for infantile hemangiomas in preterm infants receiving Darbe.

## Figures and Tables

**Table 1:** Baseline maternal and neonatal characteristics.

Parameter	No Exposure N = 262	Darbepoetin Exposure n = 92	Erythropoietin Exposure n = 36	*p* Value
Gestational Age (wk.d) ^a^	32.0 (31.0, 32.6)	28.1 (26.4, 29.2)	28.1 (27.1, 30.1)	<0.001 *
Birth Weight (g) ^a^	1630 (1435, 1780)	1020 (810, 1280)	1077 (955, 1227)	<0.001 *
Multiple pregnancy n (%)				
singleton	151 (57.6)	66 (71.7)	23 (69.4)	0.036 **
twin	111 (42.4)	26 (28.3)	10 (30.6)	
Sex n (%)				
Male	133 (50.8)	45 (48.9)	13 (38.9)	0.409
Female	129 (49.2)	47 (51.1)	20 (61.1)	
Weight for gestational age n (%)				
SGA	26 (9.9)	10 (10.9%)	10 (30.5%)	0.013 ***
AGA	229 (87.4)	81 (88.0%)	22 (66.7%)	
LGA	7 (2.7)	1 (1.1%)	1 (2.8%)	
Maternal preeclampsia n (%)	44 (16.9)	11 (11.9)	6 (19.4)	0.449
Maternal age ^a^	31 (28, 36)	34 (28.3, 37)	31 (26.5, 37)	0.27

AGA—appropriate for gestational age; SGA—small for gestational age; LGA—large for gestational age. ^a^ Median (IQR 25%, 75%). All analyses were performed by Chi-square, except for gestational age, birth weight and maternal age, which were analyzed by Kruskal–Wallis One Way Analysis of Variance on Ranks. * *p* < 0.001 in pairwise comparisons: no exposure vs. Darbe and no exposure vs. Epo; non-significant *p* in Darbe vs. Epo pairwise comparison. ** *p* = 0.023 in pairwise comparison: no exposure vs. Darbe; Non-significant *p* in the other pairwise comparisons. *** *p* = 0.003 in pairwise comparison: no exposure vs. Epo; *p* = 0.022 in pairwise comparison: Darbe vs. Epo; non-significant *p* in pairwise comparison: no exposure vs. Darbe.

**Table 2:** Short-term outcomes and hematological indices.

Parameter	No Exposure N = 262	Darbepoetin Exposure n = 92	Erythropoietin Exposure n = 36	*p* Value
Any Hemangioma n (%) 1–2 Hemangiomas ≥3 Hemangiomas	18 (6.8) ** 15 (5.7) 3 (1.1) **	17 (18.4) ** 11 (11.9) 6 (6.5) **	3 (8.3) 3 (8.3) 0	0.005 0.007
Any Hemangioma < 30 wks GA n = 175	6 (9.8) n = 61	16 (19.7) n = 81	3 (9.0) n = 33	0.158
IVH grade 1–2 IVH grade 3–4	13 (4.9) 3 (1.1) **	24 (26.1) 8 (8.7) **	4 (11.1) 1 (2.8)	<0.001
NEC n (%)	9 (3.4) ** ***	13 (14.3) **	5 (13.9) ***	<0.001
Any BPD n (%) Mild BPD Moderate BPD Severe BPD	17 (6.5) ** *** 6 (2.3) 3 (1.1) 8 (3.1) ** ***	53 (57.6) ** **** 30 (32.6) 10 (10.9) 13 (14.1) **	13 (36.1) *** **** 5 (13.9) 5 (13.9) 3 (8.3) ***	<0.001 <0.001
ROP n (%)	6 (2.3) ** ***	27 (29.3) **	7 (17.5) ***	<0.001
Bevacizumab treatment n (%)	1 (0.4) **	3 (3.3)	2 (5.5) **	0.019
FiO2 > 0.3 (d) ^a^	0 (0, 1) ** ***	2 (1, 19.5) **	1.5 (0.5, 16.5) ***	<0.001
Number of RBC transfusions ^a^	0 (0,0) ** ***	1 (0, 2) **	1 (0, 3) ***	<0.001
Number of Drug doses ^a^	0 ** ***	5.0 (3.0, 7.0) **	8.0 (5.0, 12.5) ***	<0.001
Highest Hematocrit ^a^	54.8 (49.8, 60.7) ** ***	47.7 (44.8, 52.5) **	49.9 (46.1, 54.3) ***	<0.001
Lowest Hematocrit ^a^	31 (27.8, 35.6) **	28.7 (26.8, 31) **	26.6 (25.8, 28) **	<0.001
Reticulocytes, % ^a^	1.9 (1.3, 2.9) ** ***	11 (8.7, 13.1) **	12.3 (10, 14.3) ***	<0.001
Length of stay (d) ^a^	37 (28, 46) ** ***	77 (59, 103) **	81 (62, 107) ***	<0.001

All comparisons were done by Chi-square analysis except for FiO2 > 0.3 days, number of RBC transfusions, highest and lowest hematocrit and reticulocytes, which were analyzed by Kruskal–Wallis One Way Analysis of Variance on Ranks. BPD—Bronchopulmonary dysplasia; IVH—intraventricular hemorrhage; NEC—necrotizing enterocolitis; ROP—retinopathy of prematurity. ^a^ Median (25%, 75%). **/***/**** *p* < 0.05 in pairwise comparisons between groups.

**Table 3:** Hemangioma occurrence by gestational age and drug exposure.

		23–26 Weeks	27–30 Weeks	31–32 Weeks	*p*-Value	Total
No exposure	Hemangioma n (%)	0 (0%)	6 (10%)	12 (5.9%)	0.489	18 (6.8%)
	No hemangioma	2	53	190		245
	Total	2	59	201		262
Erythropoietin exposure	Hemangioma n (%)	2 (33%)	1 (3.7%)	0 (0%)	0.051	3 (8.3%)
	No hemangioma	4	26	3		33
	Total	6	27	3		36
Darbepoetin exposure	Hemangioma n (%)	7 (25%)	9 (16.9%)	1 (9%)	0.469	17 (18.4%)
	No hemangioma	21	44	10		75
	Total	28	53	11		92
*p*-value		0.641	0.194	0.828		0.005
Total	Hemangioma n (%)	9 (25%)	16 (11.5%)	13 (6%)	0.001	38 (9.7%)
	No hemangioma	27	123	202		352
	Total	36	139	215		390

All comparisons were performed by Chi-square analyses.

**Table 4:** Within-group comparisons in exposure groups.

Parameter	Darbepoetin Exposure n = 92		*p*-Value	Erythropoietin Exposure n = 36		*p*-Value
	Hemangioma n = 17	No Hemangioma n = 75		Hemangioma n = 3	No Hemangioma n = 33	
Gestational age ^a^ wk.d	27.6 ± 1.8	28.2 ± 2	0.26	27.5 ± 2.4	28.6 ± 1.8	0.29
Birth weight ^b^, g	990 (792, 1212)	1020 (812, 1288)	0.57	968 ± 172.4	1090 ± 289	0.48
SGA n (%)	2 (11.8)	8 (10.7)	0.8	1 (33.3)	10 (30.3)	0.003
AGA	15 (88.2)	66 (88)		1 (33.3)	23 (69.7)	
LGA	0	1 (1.3)		1 (33.3)	0	
Maternal preeclampsia n (%)	2 (11.7)	9 (12)	1.0	0	7 (21.2)	1.0
Twin n (%)	4 (23.5)	22 (29.3)	0.7	1 (33.3)	10 (30.3)	1.0
Female n (%)	8 (47)	39 (52)	0.92	3 (100)	19 (57.6)	0.27
Maternal age ^b^	35 (30.7, 37)	33.5 (28, 37)	0.7	32.3 ± 6.5	31.9 ± 6.7	0.9
Number of drug doses ^b^	5 (3, 6.7)	5 (3.7, 7)	0.6	11 (10.3, 11.7)	7 (5, 13)	0.45
Day of life at 1st dose ^b^	19 (12, 23.3)	13 (10, 22)	0.17	32 (23, 37,2)	17 (11, 22.5)	0.06
Day of life at last dose ^a^	51.5 ± 16.3	50.6 ± 16.9	0.8	55 (48.2, 58)	38 (30.2, 56)	0.17
Reticulocyte count 2 weeks after 1st dose ^b^ (%)	11.1 (9, 14.6)	10.9 (8.7, 13)	0.45	11.8 ± 2.2	12.6 ± 4.5	0.77
Highest hematocrit ^a^	47.3 ± 3.7	48.9 ± 5.7	0.26	55.7 ± 1.1	49.3 ± 4.9	0.03
Lowest hematocrit ^a^	28.9 ± 3.3	28.8 ± 2.8	0.9	25.2 ± 2.3	26.9 ± 2.3	0.22
Any IVH n (%)	6 (35.2)	26 (34.6)	0.82	1 (33.3)	4 (12.1)	0.43
Grade II-VI IVH n (%)	2 (11.8)	6 (8)	0.87	0	1 (3)	
NEC n (%)	4 (23.5)	9 (12)	0.25	1 (33.3)	4 (12.1)	0.37
Days on Fio2 > 0.3 ^b^	5 (1, 24.7)	2 (0.25, 15.2)	0.12	21 (18.7, 27.2)	1 (0, 6.7)	0.06
Any BPD n (%)	11 (64.7)	42 (56)	0.7	3 (100)	10 (30.3)	0.04
Mod-Severe BPD n (%)	7 (41.2)	16 (21.3)	0.12	1 (33.3)	7 (21.2)	0.02
ROP n (%)	9 (52.9)	18 (24)	0.04	1 (33.3%)	6 (18.2)	0.48
Length of stay (d) ^a^	79 (64, 103)	75 (59, 102)	0.68	102 (98, 110)	72 (62, 93)	0.129

^a^ Mean ± SD, ^b^ Median IQR (25%,75%).

## Data Availability

The data that support the findings of this study are not openly available due to reasons of sensitivity and are available from the corresponding author upon reasonable request. Data are located in controlled access data storage at Bnai-Zion Medical Center.
